# Effects of Ultraviolet Light Supplementation on Pekin Duck Production, Behavior, and Welfare

**DOI:** 10.3390/ani10050833

**Published:** 2020-05-12

**Authors:** Gabrielle M. House, Eric B. Sobotik, Jill R. Nelson, Gregory S. Archer

**Affiliations:** Department of Poultry Science, Texas A&M University, College Station, TX 77843, USA; houseg@tamu.edu (G.M.H.); esobotik12@gmail.com (E.B.S.); jnelso15@gmail.com (J.R.N.)

**Keywords:** duck, lighting, welfare, ultraviolet, behavior

## Abstract

**Simple Summary:**

Ducks, like other poultry species, have the ability to see in the ultraviolet (UV) portion of the light spectrum; however, very little research has explored how UV lighting affects Pekin duck welfare. Therefore, the objective of this study was to determine how providing supplementary UV light in a UV-deficient environment could affect production parameters and behavior. Pekin ducks were reared under either light-emitting diode (LED) bulbs with supplemental UV light (UV) or just LED bulbs (control). The results from this study indicate that Pekin ducks reared under UV light have both lower acute and chronic stress susceptibility and lower fear responses. It was also found that eye morphology can be manipulated with UV light exposure. There were no treatment differences in production parameters such as growth, feed efficiency, tibial mineral composition, visual gait score, or stride length. The results of this study demonstrate the necessity of UV light exposure in duck growout settings and emphasize the need for correct artificial lighting in the poultry industry.

**Abstract:**

Ducks, like other domestic poultry species, can visualize the ultraviolet (UV) portion of the light spectrum; however, the importance of UV light radiation in artificially lit duck growout facilities remains unknown. The objective of this study was to determine the effects of UV light supplementation on Pekin duck production parameters, eye development, stress, and fear. Pekin ducks were reared with light-emitting diode (LED) lights supplemented with UV light or just LED lights (control). There were no differences in body weight (*p* = 0.32), feed conversion ratio (*p* = 0.38), or gait score (*p* = 0.89). Differences in eye morphology were observed, with ducks reared under UV light having narrower (12.3 ± 0.06632 mm; *p* = 0.010) and lighter (1.46 ± 0.01826 g; *p* = 0.025) eyes than the control (12.5 ± 0.05583 mm; 1.53 ± 0.02386 g). Ducks reared in UV environments had lower acute and chronic stress susceptibility with lower plasma corticosterone (6317 ± 593.79 pg/mL; *p* = 0.024), heterophil to lymphocyte ratios (0.43 ± 0.02889; *p* = 0.035), and composite asymmetry (0.58 ± 0.0298; *p* = 0.002) than control ducks (9242 ± 1120.7 pg/mL; 0.54 ± 0.04212; 0.76 ± 0.03726 mm, respectively). Ultraviolet ducks had a faster latency for the first head movement during tonic immobility (61.28 ± 9.4863 s, *p* = 0.026) and required more attempts to induce tonic immobility (1.71 ± 0.07333, *p* = 0.018) than control ducks (100.7 ± 14.846 s and 1.48 ± 0.06478, respectively). There were no differences in inversion testing (*p* = 0.91). These results indicate that UV lighting can lower stress and fear responses in Pekin ducks and can therefore increase welfare. Additionally, this study emphasizes the importance of choosing correct artificial lighting for all poultry species.

## 1. Introduction

Avian species, including ducks, have very sophisticated visual systems that allow discrimination of a broad area of the visible light spectrum [1]. Several differences in the perception of color exist between humans and birds, such as the bird’s sensitivity to the ultraviolet-A (UV) spectrum (350–400 nm) [2,3]. UV light can be perceived by birds because their ocular media is UV transparent whereas human ocular media is opaque, which does not allow UV light transmittance to the retinal cones, and due to the presence of a cone photoreceptor with a peak sensitivity for channeling short wavelengths such as those seen in the UV spectrum [3,4]. The complexity of avian visual senses provides evidence of the dependence that birds have on visual environmental cues, which consequently influences bird behavior and bird environmental interactions [5]. As poultry production systems with artificial lighting become more refined across the world, the stark differences in visual capabilities between humans and birds, especially in the context of UV light perception, must be examined further to determine optimal bird welfare under artificial lighting conditions implemented in the modern poultry industry.

Poultry use UV light to perceive and interact with their environment and conspecifics in the form of visually mediated behaviors including foraging, signaling, and social interactions [5,6,7,8]. Pekin ducks, like other poultry species, possess four cone photoreceptors sensitive to specific areas of the perceived visual spectrum. One of these retinal cones is sensitive to very short wavelengths with a maximal sensitivity at 380 nm [9]. Some poultry species form UV-reflective patches on their plumage that change and develop as the bird ages, which may be used for social signaling, individual recognition, and mate selection among conspecifics within a flock [10,11,12]. Additional UV reflectance has been observed on bedding substrates and feed in modern poultry facilities with high contrast between UV and blue wavebands [10,11]. Artificial lighting used in modern poultry houses such as fluorescent or light-emitting diode (LED) lighting contains very little UV light, which may make the UV reflective properties of plumage or the environment appear dark [13,14]. When UV supplementation is provided in otherwise UV-deficient environments as commonly seen with artificial lighting, turkey poults have been observed to be pecked less by conspecifics [15]. Additionally, a preference for environments with UV light supplementation in turkeys has been reported [16]. Although the spectral sensitivities of ducks and turkeys are similar, a study performed by Barber et al. [9] showed that turkeys are more sensitive to UV_A_ wavebands than ducks. There is evidence that mating frequency of cockerels increases when birds are in an environment supplemented with UV light, possibly due to increased cockerel locomotion, which then leads to more encounters with hens and the birds’ ability to visualize the fluorescent plumage patches of their conspecifics under UV light supplementation [6].

Stress occurs as a physiological response to environmental changes in order to reestablish homeostasis within the body [17,18]. Several measures of stress including plasma corticosterone, heterophil/lymphocyte ratios, and physical asymmetry scores of bilateral traits (such as the metatarsal length and width, and middle toe length) can be used to determine stress responses of poultry species [19,20,21,22,23]. Plasma corticosterone is a primary stress hormone released by the adrenal cortex that can be reliably used to measure poultry stress responses [19], where lower plasma corticosterone levels indicate lower stress responses [20]. Similarly, the number of heterophils in the blood will increase in response to an environmental stressor [21]. Chronic stress can affect the symmetry of physical bilateral traits [20,22]. Consequently, more physical asymmetry in bilaterally expressed traits indicate higher chronic stress in animals [23]. Poultry welfare can be assessed using tonic immobility (TI) and inversion (INV) tests, which indicate fear responses in birds [24,25,26,27]. Tonic immobility is described as a catatonic-like state in which poultry are less responsive to their surrounding environment [24]. Increased fearfulness is indicated by a longer latency to right from TI [25]. Inversion testing can be used for practical application to the commercial poultry industry, as birds are commonly inverted during handling and transport to slaughter facilities [26]. Increased wing flap intensity during inversion is indicative of higher fear response in poultry [27].

Limited research has been conducted to investigate the effects of artificial UV light supplementation on Pekin duck production and welfare. The aim of this study was to determine the effects of UV supplementation on Pekin duck production parameters, stress susceptibility, and fear response. The working hypothesis was that UV supplementation would increase production parameters such as feed conversion ratio (FCR) and would improve duck welfare by decreasing stress and fear responses.

## 2. Materials and Methods

### 2.1. Ethical Note

Ducks were managed according to the Guide for the Care and Use of Agricultural Animals in Research and Teaching [28] guidelines. All experimental methods were approved by the Texas A&M Institutional Animal Care and Use Committee (AUP #2017-0426).

### 2.2. Experimental Facilities

**Figure 1:**
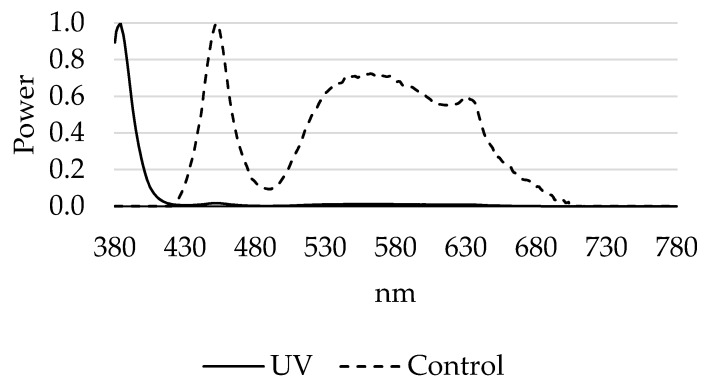
Differences in spectral power between control and ultraviolet light-emitting diode (LED) bulbs using a spectral flickering irradiance meter (SFIM-300, Everfine, Hangzhou, China).

The study was conducted using straight run Pekin ducklings (*N* = 384) obtained from Maple Leaf Farms, Inc. (Leesburg, IN) for 35 days. This experiment consisted of two treatments: control LEDs (Agrishift^®^ MLB, Once Innovations, Plymouth, MN, USA; control) and control LEDs plus ultraviolet (UV) LEDs (Agrishift^®^ HL-UVA, Once Innovations, Plymouth, MN, USA; UV). A spectral comparison of the control LED and UV bulbs are shown in Figure 1. Two replications were conducted to investigate the effects of providing supplementary UV light during the duckling growout phase on growth, stress, fear, and eye development in meat ducks.

Two light tight, tunnel ventilated rooms, each measuring 6.1 m × 9.1 m, were furnished with 8 pens (each measuring 0.9 m wide, 1.8 m long, and 0.6 m high) per room, for a total of 16 pens per trial. All pens were lined with approximately 3 inches of fresh pine shavings. Each pen also contained a nipple drinking system with 3 nipples per pen and one tube feeder, both of which could be adjusted in height during the growout period.

One of the two lighting treatments described above were used in each room. The first treatment consisted of 6 control LED light fixtures per room, which were installed directly over the pens, 3 m above the floor, and were controlled by a single dimmer and timer. The second lighting treatment consisted of (i) two ultraviolet fixtures hung 2.5 m above the floor, 3 m apart, and (ii) 6 control LED light fixtures installed as described in the first lighting treatment. To avoid “room affects”, the two lighting treatments were rotated between the two rooms upon conclusion of the first trial.

On the day of hatch, ducklings were randomly selected, weighed, and allocated to pens, where 12 ducklings were stocked in each of the 16 pens used per trial. For the first 24 h after placement, the ducks were reared under a 24L:0D photoperiod at an intensity of 20 lux as measured by light meter (SFIM-300, Everfine, Hangzhou, China) held parallel to the floor at duck head height. From days 1–10, ducks were reared under a 16L:8D photoperiod at an intensity of 20 lux. A spectral flickering irradiance meter (SFIM-300, Everfine, Hangzhou, China) was used to determine the spectral flickering irradiance of each bulb type. At 20 lux, both bulbs had a flicker index of 0.04 and 150 Hz. Beginning on day 11, lights in both treatments were dimmed to an intensity of 5 lux as per industry standards [29]. Both bulbs had a flicker index of 0.05 and 350 Hz at 5 lux. No dawn/dusk period was provided at any time point in the study.

Feed was weighed and recorded (Ohaus Champ CD-11, Pine Brook, NJ, USA), and any remaining feed at the end of each trial was subtracted from the total amount fed. Standard commercial duck starter (d0–14) and grower (d15–35) diets were provided during the course of the study. All administered feed was produced by the Texas A&M feed mill. All ducks were euthanized with a mixture of air and CO_2_ gas upon conclusion of the study on day 35 of growout.

### 2.3. Growth and Feed Conversion

To determine body weight gain, day 0 weights were subtracted from day 35 weights. Feed was weighed before being added to feeders in each pen, and residual feed was weighed back on day 15 at the end of the starter phase and again on day 35 upon conclusion of the study to allow feed intake calculations. FCR was calculated by dividing the total feed intake per pen by the total body weight gain per pen and was corrected for mortality. Mortalities were weighed and recorded daily.

### 2.4. Gait Score and Stride Length

Gait scoring was conducted at 5 weeks of age using methods described in Makagon et al. [30]. Six ducks per pen (*N* = 192) were randomly selected and placed in an observation pen with a flat concrete floor in a well-lit observation room. The gait of each duck was assessed by two trained observers with a clear view of the duck’s legs, and a single score was determined between the two observers for every duck assessed at the time of observation. A gait score was measured based on a 3-point rubric ranging from 0 to 2. A score of “0” indicated no visible waddle impediments, a score of “1” indicated slightly labored walking or limping, and a score of “2” indicated a poor gait or a reluctance to walk.

Stride length was obtained during week 5 of the study by applying black ink to the feet of all ducks assessed for gait scoring (*N* = 192) and then by allowing each duck to walk in a straight line across brown paper [31]. If a duck ran rather than walked across the paper, the test was performed again on a new piece of paper. The footprints of each duck were recorded on separate sheets of paper and allowed to dry. Stride length (cm) was analyzed by drawing a straight line beginning at the footpad of one footprint and ending at the footpad of the next footprint. A total of three lines were drawn for each duck sampled, and the average stride length for each treatment was then calculated and analyzed.

### 2.5. Tibia Bone Ash Mineral Content and Breaking Strength

On day 35, both the left and right tibia were removed from 20 randomly selected birds per treatment (*N* = 80). Twenty birds per treatment (*N* = 80) were randomly selected and euthanized via a mixture of air and CO_2_ gas on d 35. The heads of all euthanized ducks were removed and placed in bags of deionized water to be stored overnight. The muscle, connective tissue, and fibula were removed from each tibia, and the bones were dried using a Forced Air Oven (VWR 89511-410, Radnor, PA) at 100 °C for 12 h. Left tibias were used to measure bone mineral content. Right tibias were used to measure bone breaking strength. Left tibias were defatted using diethyl ether for 6–8 h and air dried under a chemical hood, allowing all remaining ether to evaporate. Defatted tibias were then dried again at 100 °C for 12 h and then ashed at 600 °C in ceramic crucibles for 24 h. To determine bone mineral content, all tibias and crucibles were weighed before and after ashing. To minimize moisture content, crucibles were kept at 100 °C for 12 h prior to ashing. Right tibia breaking strength (g) was determined using a QC-SPA system (TSS, York, England) to break each tibia bone at the center point of the tibial shaft.

### 2.6. Eye Development

The physical differences in eye development between treatments were evaluated during the fifth week of growout. The same 20 birds per treatment (*N* = 80) used for tibia bone ash mineral content and breaking strength were used to determine all eye development parameters. After euthanasia on d 35 (described above), the heads of all euthanized ducks were removed and placed in bags of deionized water to be stored overnight. The following day, the left and right eyes from each duck were extracted and measured. A calibrated Craftsman IP54 Digital Caliper (Sears Holdings, Hoffman Estates, IL, USA) was used to measure the side-to-side diameter (mm) and back-to-front diameter (mm) of each eye. Additionally, the weight of each eye (g) was recorded. The average eye length, width, weight, and the differences between the left and right eyes for each of these respective measurements was recorded for each treatment.

### 2.7. Stress Susceptibility Measures

#### 2.7.1. Plasma Corticosterone and Heterophil to Lymphocyte Ratios

At 35 days of age, 20 ducks per treatment (*N* = 80) were randomly selected for plasma corticosterone (CORT) and heterophil to lymphocyte ratio (HL) analysis. Approximately 1–2 mL of blood was collected from the brachial wing vein of each duck. A small drop of blood from each bird sampled was smeared on a glass plate for heterophil to lymphocyte ratio (HL) analysis (described below). The remaining blood collected was injected into a plasma separation gel and lithium heparin vacutainer (BD 368056, BD, Franklin Lakes, NJ, USA) and temporarily stored on an ice bath. Once all blood samples had been collected, all vacutainers were spun down using a centrifuge (Eppendorf 5804, Eppendorf North America, Hauppauge, NY, USA) at 4000 RPM for 15 min to separate the plasma and blood cells. Each blood plasma sample was then poured into a 2-mL microcentrifuge tube and stored at −19 °C until further analysis was performed. A hematology staining kit (Cat# 25034, Polysciences Inc, Warrington, PA, USA) was used for staining blood smear slides used for HL.

The plasma corticosterone concentration from each sample was analyzed using a commercially available ELISA kit (Enzo Life Sciences, ADI-901-097, Farmingdale, NY, USA). The inter- and intra-assay %CV were both under 5%.

To determine the heterophil to lymphocyte ratio of collected samples, one layer of stained blood cells on glass slides were observed under 40× magnification using an oil immersion lens on a microscope (Omax DCE-2, Kent, WA, USA). The number of heterophils and lymphocytes observed in an area of the blood smear slide without overlapping cells was counted using a keystroke counter (SEOH B4001-5LC, Navasota, TX, USA) until a total of 100 cells had been recorded [32]. Increased plasma corticosterone [33] and heterophil to lymphocyte ratios [21] indicate higher stress susceptibility in poultry.

#### 2.7.2. Physical Asymmetry

On day 35 of the current study, 60 live ducks per treatment (*N* = 240) were randomly selected to be measured for physical asymmetry of 3 bilateral traits (ASYM) as described in Archer et al. [34]. A calibrated Craftsman IP54 Digital Caliper (Sears Holdings, Hoffman Estates, IL) was used to measure the metatarsal length (ML), metatarsal width (MW), and middle toe length (MTL) of the left and right legs of each duck. The composite asymmetry score for each duck was calculated by taking the sum of the absolute value of the left measurement subtracted from the right measurement of each trait and then by dividing by the total number of traits [35].

### 2.8. Fear Response Measures

#### 2.8.1. Tonic Immobility

Tonic immobility measurements were collected during the fifth week of growout using methods described in Archer [35] and House et al. [36]. The described methods were designed for the induction of TI in broilers; therefore, the methods used in the current study were slightly modified by lengthening the time for which pressure was applied to the thoracic cavity to induce TI, as described below. Sixty ducks per treatment (*N* = 240) were randomly selected and were gently placed on their back in a wooden U-shaped cradle lined with black cloth. Slight pressure was placed on the thoracic cavity of the duck for approximately 25 s, until tonic immobility was induced. Contact was then removed, and a timer was started. Each duck must be immobile for at least 10 s in order for its latency to right from TI to be recorded. If the duck righted itself in under 10 s, it was recorded as a time of 0 s. Otherwise the first head movement and the duck’s latency to right itself was recorded, with a maximum time of 10 min. Each duck was allowed a maximum of 3 attempts to be successfully tested. If all 3 attempts were unsuccessful, the duck’s final time was recorded as 0 s. A longer latency to right during tonic immobility is indicative of greater fear responses in poultry [24].

#### 2.8.2. Inversion

Inversion measurements were also collected during the fifth week of growout. Sixty ducks per treatment (*N* = 240) were randomly selected and caught and then inverted while holding each duck by its legs until the duck ceased to wing flap or for 30 s [34]. Each duck inversion was recorded for later observation (Cannon, ZR900, Melville, NY, USA; 24 frames per second). The number of wing flaps and the duration of wing flapping during inversion for each duck was recorded by a trained video observer, and wing flap intensity was determined by dividing the number of wing flaps by the duration of wing flapping during inversion. Greater intensity of wing flapping indicates higher fear responses in poultry species [26].

## 3. Statistical Analysis

General Linear Models (GLM) were used to investigate the treatment, trial, and treatment × trial effects on feed conversion, weight gain, eye parameters, plasma corticosterone concentration, heterophil to lymphocyte ratio, composite asymmetry scores of bilateral traits, tonic immobility, and inversion. Levene’s test for homogeneity of variance and the Shapiro–Wilk test for normality were used to test all GLM assumptions. All assumptions were met without transformations, and all planned comparisons were tested using the least significant difference test. Windows SAS 9.3 (SAS Institute Inc., Cary, NC, USA) was used to perform all analyses. *p* < 0.05 was used to determine all significant differences, and only treatment effects were discussed in the current study, as there were no effects observed on trial or treatment × trial interaction.

## 4. Results

All results for FCR, d 35 body weight, gait parameters, tibia bone ash mineral content, and tibia bone breaking strength are shown in Table 1. No differences between lighting treatments were found in body weight or FCR (*p* > 0.05). Additionally, no differences in gait score or stride length (*p* > 0.05) were observed between the two treatments, as shown in Table 2. No differences were found in the percent tibia bone ash mineral content or tibia bone breaking strength (*p* > 0.05).

The UV treatment had lighter eyes (1.46 ± 0.01826 g; *p* = 0.025) and narrower eyes (12.3 ± 0.06632 mm; *p* = 0.010) than the control treatment (1.53 ± 0.02386 g and 12.5 ± 0.05583 mm, respectively). However, there was no difference in average eye length between the two treatments (*p* > 0.05). Additionally, the average differences in weight, width, and length between the left and right eyes of each duck did not differ between the two treatments (*p* > 0.05). All data for eye development parameters are shown in Table 2.

All results for stress susceptibility and fear response parameters are shown in Table 3. The UV ducks had lower plasma corticosterone concentrations (6317 ± 593.79 pg/mL; *p* = 0.024) and lower heterophil to lymphocyte ratios (0.43 ± 0.02889; *p* = 0.035) compared to control ducks (9242 ± 1120.7 pg/mL and 0.54 ± 0.04212, respectively), indicating lower stress levels in ducks reared under supplemental UV light. The UV ducks had lower composite asymmetry scores (0.58 ± 0.0298; *p* = 0.002) than control ducks (0.76 ± 0.03726 mm), indicating lower long-term stress in the UV treatment compared to the control treatment.

Ultraviolet ducks had a faster latency for the first head movement during tonic immobility (61.28 ± 9.4863 s, *p* = 0.026) and required more attempts to induce tonic immobility (1.71 ± 0.07333, *p* = 0.018) than control ducks (100.7 ± 14.846 s and 1.48 ± 0.06478, respectively). There were no differences in inversion intensity or the latency to right during tonic immobility between the two treatments.

## 5. Discussion

### 5.1. Growth and Production Parameters

In the current study, there were no differences in duck growth performance, feed conversion, gait score, stride length, tibia bone ash mineral content, or tibia bone breaking strength. A study previously conducted by Zhang et al. [37] found that broiler chickens reared under incandescent light supplemented with UV light had higher body weight, feed conversion, and skeletal development compared to those reared in an ultraviolet deficient environment, which could be attributed to higher levels of phosphorous, calcium, and growth hormone in birds reared under UV supplementation. No differences in feed conversion ratios or body weight were observed in broiler chickens exposed to either UV-deficient lighting or lighting containing UV supplementation [38]. To our knowledge, no studies have examined the effects of ultraviolet light on Pekin duck growth and bone development. It has been observed that ducks have a lower spectral sensitivity to the ultraviolet spectrum than other poultry species [9], making species comparisons between broiler chickens and Pekin ducks difficult. Therefore, it may be possible that production parameters and bone development of Pekin ducks may not reflect that of broilers when both species are reared under UV light, which may explain why no differences in production parameters or bone development were observed in the current study.

### 5.2. Eye Development

In the current study, ducks reared under supplementary UV light had lighter and narrower eyes than control ducks. No differences were observed in eye width or average differences between the length, width, or weight of the left and right eyes of each duck. Very few studies have examined the effects of ultraviolet light on eye development in poultry. A study conducted by Hogsette et al. [39] compared the eye pathology of hens exposed to blacklight-blue or blacklight fly trap lamps; however, no differences in eye morphology were observed. To our knowledge, no other studies have focused on poultry eye development under UV light conditions, and further research is needed to determine how changes in eye anatomy and weight affect the welfare of Pekin ducks and other poultry species.

### 5.3. Stress Susceptibility

Ducks reared under UV supplementation had lower plasma corticosterone concentrations, lower heterophil to lymphocyte ratios, and grew less asymmetrically than those reared in UV-deficient environments. It has been previously demonstrated that stress parameters such as CORT, H:L, and ASYM can be affected by light [27,32,34,40,41,42]. The biological functions and development of poultry and other animals can be disrupted by stress [43]. A study performed by Huth and Archer [20] found that two different LED bulbs lowered CORT, H:L, and ASYM in chickens, indicating lower stress susceptibility in chickens reared under two particular LED spectrums compared to a compact fluorescent lamp (CFL) bulb. Furthermore, both broiler [36] and laying hens [42] had lower stress susceptibility when reared under LEDs supplemented with UV light, as indicated by lower CORT, H:L, and ASYM than birds reared under just LEDs. The results of this study provide additional evidence, suggesting that UV supplementation can alter both acute and chronic stress susceptibility in ducks.

### 5.4. Fear Responses

No differences were found in the latency to right from tonic immobility or in wing flap intensity between UV and control ducks in this study, although UV ducks had a faster latency for first head movements during tonic immobility and also required more attempts to induce tonic immobility. Tonic immobility can be divided into two stages, with the first stage being complete immobility without head movements and the second stage involving head movements [44], meaning head movement may be associated with the latency to right from TI. A shorter latency to right from TI was observed in broilers subjected to UV light instead of just LED lighting [36,45]. Sobotik et al. [42] also saw a shorter latency to right in laying hens housed under LED bulbs supplemented with UV light. Laying hens and broiler chickens have also been shown to have lower wing flap intensities when reared under UV light supplementation [36,42]. The results from the current study suggest that, although there is no difference in the latency to right from TI or INV wing flap intensity between treatments, there is a possibility that ducks do experience the first stage of tonic immobility (complete immobility without head movements) [44] for a shorter duration than control ducks. Although UV ducks and control ducks did not differ in the latency to right from TI, it is a possibility that UV ducks are more inquisitive about their environment even while experiencing extreme amounts of fear due to the ability to more effectively perceive the UV reflectivity of objects and may therefore experience shorter durations of the first stage of TI as a result.

Although there is very little knowledge concerning the effects of lighting on Pekin ducks in growout settings, as the meat duck industry grows within the United States and abroad, there will continue to be an increasing and persistent need for better welfare in Pekin duck growout facilities. Rearing calmer birds will ultimately result in fewer mortalities during transport and a fewer damaged carcasses during processing [46]. Based on the results of this study, it can be concluded that ducks reared under UV light supplementation have increased welfare compared to ducks reared in UV-deficient environments, as represented by the behavioral tests and stress susceptibility measures described above.

## 6. Conclusions

In comparison to other aspects of poultry production such as rearing parameters, biosecurity, and nutritional requirements, knowledge of poultry light perception and the effects of lighting on poultry behavior and development is relatively limited [47]. Similar to other poultry species, ducks utilize the ultraviolet portion of the light spectrum for visually mediated behaviors, resulting in more effective environmental perception under ultraviolet light supplementation than in environments with UV-deficient lighting. However, the spectral sensitivity of ducks is different from other poultry species [9], making it critical to determine the effects of UV exposure on the performance and welfare of Pekin ducks. Based on the results of the current study, Pekin ducks reared under an environment illuminated with both LED bulbs and UV light supplementation have decreased stress and fear responses, indicating better welfare than ducks reared under only LED bulbs, which are deficient in UV light. Eye development can be manipulated by the presence of ultraviolet light; however, the welfare impacts of these changes in development are unknown and present future research opportunities. These results continue to emphasize the need for correct light spectrum supplementation in an artificially illuminated poultry houses.

## Figures and Tables

**Table 1:** A comparison of results for growth, gait, and tibia bone quality parameters of Pekin ducks reared under normal LED (control) or control lighting supplemented with ultraviolet light (UV) in two replicative studies.

Treatment	FCR ^1^	D 35 Body Weight ^1^	Gait Score ^2^	Stride Length ^2^	Tibia Bone Ash ^3^	Tibia Breaking Strength ^3^
		kg		cm	%	g
UV	1.66	2.70	0.10	18.4	45.7	33,239
Control	1.57	2.62	0.083	18.5	45.8	32,320
SEM	0.03832	0.03742	0.0211	0.2249	0.230	1459.39
*P*-Value	0.38	0.32	0.89	0.90	0.79	0.53

Abbreviations: FCR, feed conversion ratio; ^1^ *N* = 32 pens; ^2^ *N* = 192 birds; ^3^ *N* = 80 birds.

**Table 2:** A comparison of results for eye development parameters of Pekin ducks reared under normal LED (control) or control lighting supplemented with ultraviolet light (UV) in two replicative studies.

Treatment	Eye Weight	Eye Length	Eye Width	Abs. Eye Weight	Abs. Eye Length	Abs. Eye Width
	g	mm	mm	g	mm	mm
UV	1.46	8.14	12.3	0.058	0.31	0.36
Control	1.53	8.23	12.5	0.070	0.42	0.32
SEM	0.01541	0.02753	0.04494	0.01183	0.02970	0.03469
*P*-Value	0.025	0.10	0.010	0.60	0.065	0.57

Abbreviations: Abs., absolute value of the difference between left and right eyes of all ducks sampled; ^1^ *N* = 80 birds.

**Table 3:** A comparison of results for stress susceptibility and fear response parameters of Pekin ducks reared under normal LED (control) or control lighting supplemented with ultraviolet light (UV) in two replicative studies.

				Tonic Immobility ^2^			
Treatment	Corticosterone ^1^ pg/mL	Heterophil to Lymphocyte Ratio ^1^	Composite Asymmetry Score ^2^ mm	Latency to Right s	First Head Movement s	# Attempts	Inversion ^2^ Flaps/s
UV	6,317	0.43	0.58	157.69	61.28	1.71	2.85
Control	9,242	0.54	0.76	202.96	100.7	1.48	2.83
SEM	651.24	0.0261	0.0245	12.4874	8.8824	0.05055	0.07586
*P*-Value	0.024	0.036	0.0002	0.070	0.026	0.018	0.91

^1^*N* = 80 birds; ^2^ *N* = 240 birds.
